# Biophysical Analysis of Lipopolysaccharide Formulations for an Understanding of the Low Endotoxin Recovery (LER) Phenomenon

**DOI:** 10.3390/ijms18122737

**Published:** 2017-12-16

**Authors:** Wilmar Correa, Klaus Brandenburg, Ulrich Zähringer, Kishore Ravuri, Tarik Khan, Friedrich von Wintzingerode

**Affiliations:** 1 Forschungszentrum Borstel, Leibniz-Zentrum für Medizin und Biowissenschaften, Parkallee 1-40, D-23845 Borstel, Germany; wcorrea@fz-borstel.de (W.C.); uzaehr@web.de (U.Z.); 2 Brandenburg Antiinfektiva GmbH, Parkallee 10b, D-23845 Borstel, Germany; 3 Pharmaceutical Development & Supplies, F. Hoffmann-La Roche Ltd., 4070 Basel, Switzerland; satya_krishna_kishore.ravuri@roche.com (K.R.); tarik.khan@roche.com (T.K.); 4 Roche Diagnostics GmbH, Nonnenwald 2, Roche Diagnostics GmbH, 82377 Penzberg, Germany; friedrich.von_wintzingerode@roche.com

**Keywords:** endotoxin, lipopolysaccharide, Low Endotoxin Recovery, phase transitions, polysorbate, LPS aggregates, Small Angle X-ray Scattering, MAT, LAL and LER

## Abstract

Lipopolysaccharides (LPS, endotoxin) are complex and indispensable components of the outer membrane of most Gram-negative bacteria. They represent stimuli for many biological effects with pathophysiological character. Recombinant therapeutic proteins that are manufactured using biotechnological processes are prone to LPS contaminations due to their ubiquitous occurrence. The maximum endotoxin load of recombinant therapeutic proteins must be below the pyrogenic threshold. Certain matrices that are commonly used for recombinant therapeutic proteins show a phenomenon called “Low Endotoxin Recovery (LER)”. LER is defined as the loss of detectable endotoxin activity over time using compendial *Limulus* amebocyte lysate (LAL) assays when undiluted products are spiked with known amount of endotoxin standards. Because LER poses potential risks that endotoxin contaminations in products may be underestimated or undetected by the LAL assay, the United States (U.S.) Food and Drug Administration’s (FDA’s) Center for Drug Evaluation and Research (CDER) has recently started requesting that companies conduct endotoxin spike/hold recovery studies to determine whether a given biological product causes LER. Here, we have performed an analysis of different LPS preparations with relevant detergents studying their acyl chain phase transition, their aggregate structures, their size distributions, and binding affinity with a particular anti-endotoxin peptide, and correlating it with the respective data in the macrophage activation test. In this way, we have worked out biophysical parameters that are important for an understanding of LER.

## 1. Introduction

Lipopolysaccharides (LPS), the endotoxins of most Gram-negative bacteria, belong to the strongest immune-stimulating compounds known in nature. This property may be beneficial at low concentrations, but pathophysiological at high concentrations, leading to severe sepsis and septic shock with high lethality [1]. Since LPS is a constituent of nearly all Gram-negative bacteria, it is a ubiquitous contaminant. It is well-known that the lipid A part of LPS is its “endotoxic principle”, which for most relevant bacterial species, such as *Escherichia coli* and *Salmonella* spp. consists of a bisphosphorylated diglucosamine backbone to which six acyl chain residues are linked [1]. There are also lipid A with underacylated lipid A parts (tetra or pentaacyl) with low biological activity [1], which, however, are not relevant in the context of this investigation.

LPS is able to elicit severe safety risks even at very low concentrations. Thus, in clinical studies of Opal and co-workers, it was found that sepsis patients belonging to the survivors had a medium LPS serum concentration of 0.3 ng/mL and the non-survivors 0.7 ng/mL [2]. The reason for this is the fact that LPS induces a “cytokine storm” (interleukins, tumor-necrosis-factor-α (TNF-α) and many others), leading to a septic shock. Therefore, it is of uttermost importance to control LPS-load, in particular, in parenteral pharmaceutical formulations, such as recombinant therapeutic proteins, which are anticipated to be injected.

Low Endotoxin Recovery (LER) poses potential risks that endotoxin contaminations in products may be underestimated or undetected by the *Limulus* amebocyte lysate (LAL) assay.

There are various publications in recent years dealing with this subject, in most cases giving no coherent explanation for the occurrence of the effect [3,4]. As one possible explanation, it was proposed that the presence of certain buffers and detergents, in most cases citrate and polysorbate 20 and 80, leads to a drastic disaggregation of LPS down to a monomeric form, which was found to represent an inactive form in the LAL assay, as well as the macrophage activation test (MAT) [5]. Since there are also papers that are indicative of an active monomeric form of LPS [6], and since the assumption of a monomeric form induced by the detergents could not be verified directly to date, we had a closer biophysical look on the phenomenon. Regarding the biological techniques to prove the presence of LPS, usually the *Limulus* amebocyte lysate (LAL) assays in different modifications (gel clot, chromogenic, turbidimetric) and the recombinant Factor C assay are used, which are all based on the interaction of factor C of the Limulus cascade with LPS [7]. An alternative method is the determination of the LPS-induced stimulation of human cells, such as mononuclear cells (MNCs, monocytes, or macrophages (generally called MAT test)) [8]. It must be noted that both techniques suffer from disadvantages: For activating the LAL test, the structural presence of only a LPS part structure is necessary, i.e., the 4′-phosphate diglucosamine backbone of lipid A [8] in acylated form. Therefore, a LAL signal is already seen with underacylated lipid A structures (tri-, tetra-, and pentaacyl groups), which, in the human immune system, do not or only to a small degree elicit an inflammation reaction [9]. Furthermore, the LAL assay can also be activated by β-d-glucans. The disadvantage of the MAT lies in the fact that it reacts to all of the bacterial immune-stimulating toxins, i.e., also to those from Gram-positive origin, which has been shown to result mainly from lipoproteins and/or their shortened lipopeptide variants [10].

In the present work, we have applied a variety of biophysical techniques to gain more insight into the LER phenomenon and to characterize a possible influence of different detergent formulations on (i) the gel to liquid crystalline phase transition behavior of different LPS, (ii) their aggregate structure, and (iii) aggregate sizes. Furthermore, the action of a recently well-described antimicrobial peptide Aspidasept^®^ [11,12] on the binding to LPS in different formulations was studied.

These data were directly correlated to their activity in the MAT. In this way, we should be able to better understand the influences of the different parameters on LER.

## 2. Results and Interpretations

### 2.1. Gel to Liquid Crystalline Phase Transition of the Acyl Chains

**Figure 1:**
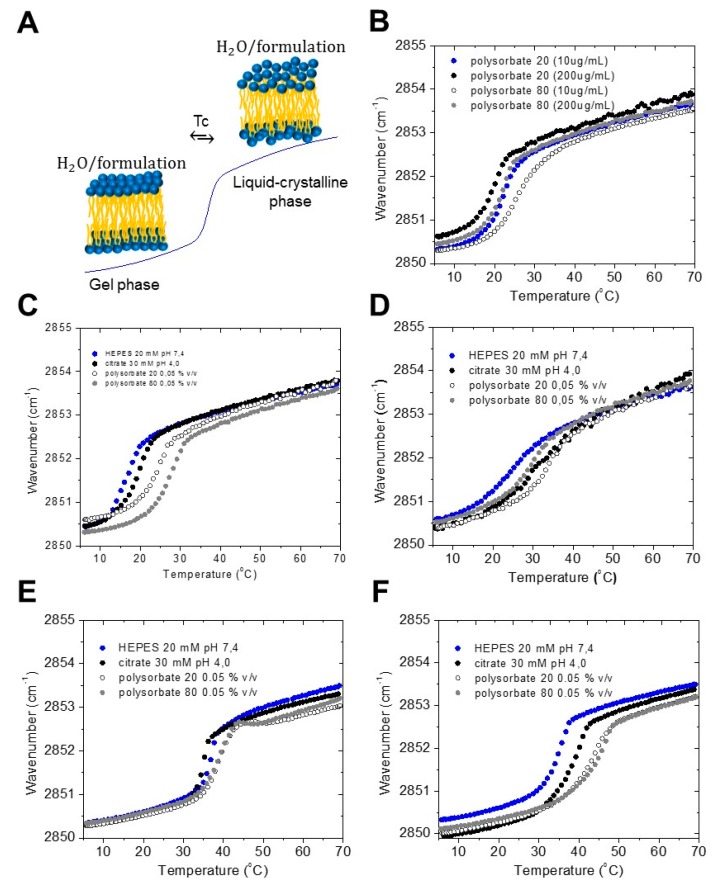
Effect of the formulation on the gel to liquid crystalline phase transition of lipopolysaccharides (LPS). (**A**) Schematic representation of the gel to liquid-crystalline phase transition of a lipid bilayer. Phase transitions of (**B**) LPS from *E. coli* O111:B4 in two different concentration of polysorbate 20 and 80; (**C**) LPS from *E. coli* O111:B4 in different formulations; (**D**) LPS from *E. coli* 055:B5 in different formulations; (**E**) LPS from *S. minnesota* Rb mutant strain R345 in different formulations; and, (**F**) LPS from *S. minnesota* Rd-mutant strain R7 in different formulations.

All amphiphilic compounds, such as LPS, can adopt two states of order of the acyl chains, one highly ordered (gel) with relatively rigid chains at lower temperatures, and one unordered (liquid-like) with highly fluid chains at higher temperatures, Figure 1A. Typically, the phase transition temperature T_c_ of enterobacterial LPS is around 30 to 37 °C, i.e., close to the physiological temperature [13]. For the different smooth form LPS, however, due to the heterogeneous LPS mixtures, differing in the degree of acylation and length of the saccharide chains, frequently lower T_c_ values may be observed. This parameter might be of importance for the ability of the compounds to interact with target structures. Fourier-infrared spectroscopy (FTIR) is the method of choice, by monitoring the symmetric stretching vibrational band at 2850–2853 cm^−1^ of the methylene groups, with the former value characteristic for the gel and the latter for the liquid crystalline phase. In the following, selected commonly used LPS from wild-type strains from *E. coli* O55:B5 and *E. coli* O111:B4 (Sigma, Deisenhofen, Germany), LPS Rb and Rd mutants from *Salmonella minnesota* R345 and *Salmonella minnesota* R7 (own purified samples), respectively, in different buffers and detergents were analysed. The data are shown in Figure 1B–F. As can be seen, the values of T_c_ are sensitively dependent on the different formulations, with values of approximately 17 °C for LPS O111:B4 in buffer, and increasing values up to 27 °C for the polysorbate 80 (at concentrations below the critical micellar concentration (CMC)) preparation. Interestingly, when the polysorbate concentrations are increased to values higher than the CMC, then the transition values considerably decrease (Figure 1B).

The measurements for LPS O55:B5 show a very broad phase transition range, which is indicative of a very heterogeneous mixture of this smooth form LPS (Figure 1C). Again, the transition value is lowest (appr. 25 °C) for the buffer system, but in this case, highest for the polysorbate 20 sample. In Figure 1B, the phase transition behavior is measured at two polysorbate concentration (10 and 200 μg/mL), representing values below and above their respective critical micellar concentration (CMC). It can be seen that there is a decrease of the T_c_ value at the higher polysorbate concentrations. Furthermore, there is an increase of the T_c_ value in the sequence citrate, polysorbate 20, and polysorbate 80.

Since it has been found that the bioactive form within the heterogeneous wild-type strains corresponds to R-mutants (Ra- or Rb-mutants as found in [14]), two of them were also investigated. The data for the Rb-mutant LPS from *S. minnesota* (Figure 1E) clearly show a much smaller and sharp transition range, according to the fact that this LPS is homogenous and pure. The T_c_-values are lowest for the citrate and are highest for the polysorbate formulations, and corresponds to previous findings with values around 35 to 37 °C [15]. The results for a LPS Rd from *S. minnesota* strain R7, which has the lowest T_c_ (ca. 34 °C) in buffer due to the short oligosaccharide chain, are presented in Figure 1F. Surprisingly, there is a stronger increase in the phase transition temperature, as seen for the other LPS samples.

Summarized, two tendencies are observed: for the two polysorbates, the concentration above their CMCs (200 μg/mL) lead to a lower phase transition temperature of LPS. For the citrate and polysorbate formulation below CMC (10 μg/mL) there is an increase in transition temperature as compared to the HEPES control. The latter effect corresponds to an increase of the rigidity of the hydrocarbon chains, and with that, of the whole LPS assembly.

### 2.2. Aggregate Structures of LPS Preparations Used in This Study

**Figure 2:**
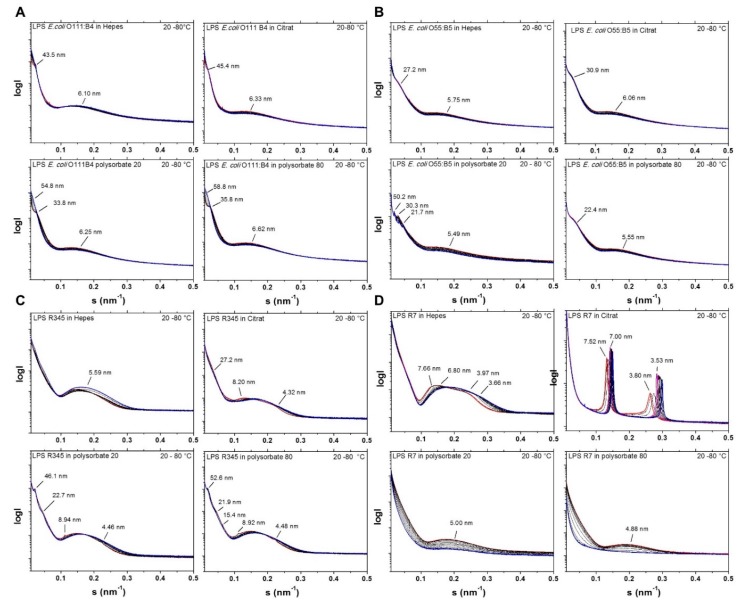
Effect of the formulation on the supramolecular LPS aggregate structure. Synchrotron radiation small-angle X-ray scattering patterns of LPSs from (**A**) *E. coli* O111:B4; (**B**) *E. coli* O55:B5; (**C**) *S. minnesota* Rb strain R345; and, (**D**) *S. minnesota* Rd strain R7. The logarithm of the scattering intensity is plotted versus the scattering vector s ($s=\frac{1}{d}$, with d being the spacings of the reflections).

The aggregate structure of LPS was described as important parameter, which determines the property of these amphiphilic compounds to exhibit biological activity [16,17]. For this, small-angle X-ray scattering (SAXS) via synchrotron radiation was applied, since under near physiological conditions (high water content) laboratory X-ray sources are not sufficient due to lack in brilliance. In the Figure 2, the LPS samples described above were measured in the temperature range 20 (blue line) to 80 °C (red line). Presented are the logarithm of the scattering intensity log I versus the scattering vector s ($s=\frac{1}{d}$, d = spacings of the reflections). The data for LPS from *E. coli* O111:B4 (Figure 2A) exhibit a complex scattering pattern, which is characteristic for a bilayered structure in the s-value range 0.1 to 0.35/nm (reflection centered at 6.0 to 6.3 nm), and a cubic periodicity (reflections at 40 to 56 nm). Interestingly, the polysorbate formulations show changes in the aggregate structure, which is obviously correlated with the phase transitions temperature. This can be deduced from the jump of the reflections at 33.8 and 35.8 nm to values above 50 nm. For LPS *E. coli* O55:B5 (Figure 2B), two main reflections around 5 to 6 nm and 20 to 30 nm are seen. In the case of the polysorbate 20 formulation, there is a complex reflection pattern between 20 and 50 nm. Also, in the case of LPS Rb from *S. minnesota* R345, the situation is similarly complex (Figure 2C). The LPS in HEPES indicates unresolved spectra, the LPS in citrate, and polysorbate indicate a higher degree of order, by showing multilamellar-like reflections at 8.20 and 4.32 nm (citrate), 8.94 and 4.46 nm (polysorbate 20), and 8.92 and 4.48 nm (polysorbate 80). Moreover, the polysorbate formulations exhibit reflections at around 20 to more than 50 nm. The observation is different for LPS Rd mutant (Figure 2D). The patterns for the sample in HEPES already exhibit some weak scattering maxima at 7.66 and 3.97 nm, which can be assigned to a multilamellar arrangement. This is strongly expressed for the LPS in citrate, in which at the lowest temperature peaks are clearly seen at 7.52 and 3.80 nm, 1st and 2nd order of a multilamellar aggregate, which shift to higher values at the higher temperature due to acyl chain melting (Figure 1E). Interestingly, no sharp reflections are seen for this LPS in the polysorbate formulations, but scattering intensity is seen in the s-value range 0.13 to 0.35/nm.

Summarized, for the wild type LPS from *E. coli* O111:B4 as well as Rb-mutant from *Salmonella minnesota* R345, the scattering patterns clearly indicate a complex change of the aggregate structures in the polysorbate containing chelating buffers as compared to HEPES and citrate formulations alone, lacking the detergents. For a forward assessment, it should be noted that the multilamellar structures that are seen here for LPS Rb and LPS Rd correspond to the bio-inactive structures of LPS [15,17,18].

### 2.3. Thermodynamics of Binding of the Synthetic Anti-LPS Peptide (SALP) Pep19-2.5 with the Different LPS Preparations

It has been reported that particular antimicrobial peptides (AMP) from the SALP (synthetic anti-LPS peptides) series, compound Pep19-2.5, binds and neutralizes LPS very efficiently [11,19]. This peptide is a 20’mer and consists of a N-terminal region with charged and polar amino acids and a C-terminal region with essentially hydrophobic amino acids. It is scheduled to fight against severe infections, such as sepsis [11]. The binding of the peptide to LPS preparations was tested here because it is known that the lipid A backbones, in particular the lipid A phosphates, are targets for the peptides, which is important with respect to the biological assay: Cellular activation in the MAT runs via the binding of the bisphosphorylated lipid A backbone to the TLR4 receptor.

To test the neutralizing activity of Pep19-2.5 with the different LPS formulations, isothermal titration calorimetry (ITC) was applied. The enthalpy change of this interaction can give information about the kind of binding process, which may be of exothermic or endothermic nature, or a mixture of both, with which the driving force of the interactions can be determined. In the experiments, in a first step all of the compounds were dissolved in the respective formulations, and in a second step the peptide was dissolved in water and was then added to the different formulations.

The data (Figure S1, Table 1) show similar binding characteristics, only for the polysorbate 20 formulation at 200 μg/mL there is an increase of the saturation curve to higher Pep19-2.5:LPS molar ratio values. Furthermore, the data for the peptide dissolved in water and then dispersed into the LPS formulations indicates a lower binding enthalpy of 40–50 kJ/mole at the beginning of the titration.

From the Figure S1A–C, the thermodynamic parameters can be calculated as presented in Table 1. The ITC results show that the basic neutralization mechanisms of LPS by Pep19-2.5 remain similar for all of the different formulations. There are some variations of the initial enthalpy change ∆H and the saturation values as indicated, with the values of LPS in HEPES buffer at −67 kJ/mole and saturation value *n* = 0.245 exhibiting highest affinity and LPS in polysorbate 80 (10 μg/mL) at −43 kJ/mole and *n* = 0.4 exhibiting lowest affinity.

To summarize, the findings for the LPS representing different *O*-serotypes and formulations are indicating similar neutralizations mechanisms, which is a matter of fact that the peptide essentially binds to the lipid A part of LPS, its “endotoxic principle”. For all bioactive LPS, the lipid A part consists of a hexaacylated diglucosamine moiety phosphorylated in positions 1 and 4’. Surprisingly, the neutralization (saturation) of LPS takes place at higher peptide concentrations for the polysorbate formulations.

### 2.4. Stimulation of Immune Cells by the LPS in the Different Formulations (MAT)

**Figure 3:**
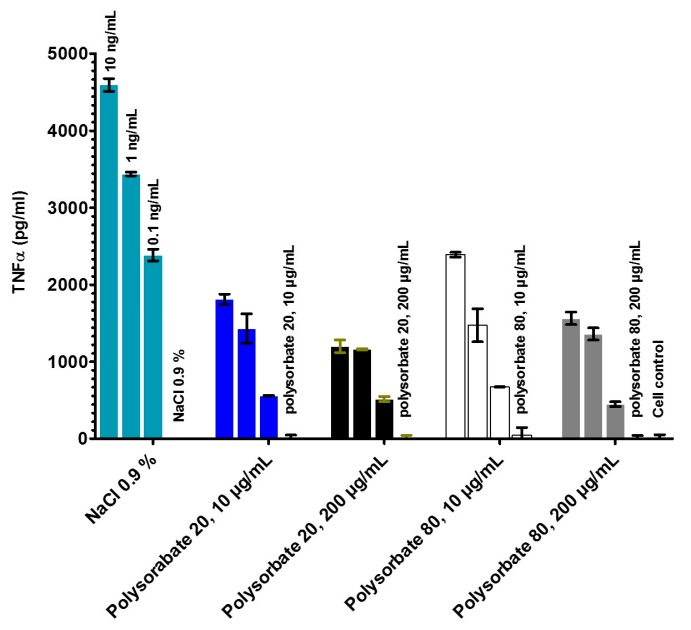
Secretion of tumor-necrosis-factorα (TNF-α) by human mononuclear cells induced by LPS from *E. coli* O55:B5. LPS aggregates were prepared in NaCl 0.9% and polysorbate 20 and 80. Three LPS concentrations 10, 1 and 0.1 ng/mL were tested. The error bar comes from two-fold determination of TNF-α concentration in the ELISA.

The immune-stimulating activity of human mononuclear cells by compounds can be tested in an ELISA (MAT), for which TNF-α as sensitive cytokine is selected, which is secreted by the cells already after some hours. In a first step, the LPS from *E. coli* O55:B5 was tested in different formulations, NaCl 0.9%, polysorbate 20 and polysorbate 80, each at two concentrations (10 and 200 μg/mL), see Figure 3. As can be seen, the TNF-α secretion is highest for the sample in NaCl, whereas the highest concentrations of the two polysorbate samples lead to a strong reduction of the activity, with polysorbate 20 having the strongest influence on the reduction.

**Figure 4:**
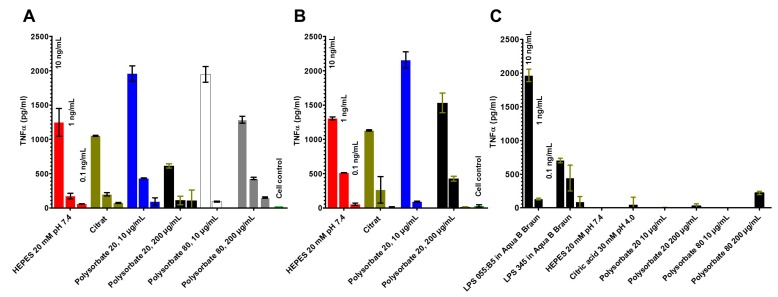
LPS-induced secretion of TNF-α by human mononuclear cells with different LPS formulations. LPS from *E. coli* O55:B5 (**A**), LPS R345 (**B**), and buffers and LPS control dissolved in water (**C**). Stimulation of human mononuclear cells was made at the three concentration: 1.0, 0.1 and 0.01 ng/mL, and the activity is recalculated. The error bar results from twofold measurement of TNF-α in the ELISA.

To examine also the dependence on the LPS chemotypes, further stimulation data were obtained by investigating LPS O55:B5 and LPS Rb mutant R345 in different formulations (Figure 4A–C). The data indicate differences in particular for the polysorbate formulations.

### 2.5. Measurements of the Size Distribution of LPS Aggregates by Zeta Sizer

Aggregate sizes and their distributions has been discussed as a parameter, which influences LER [3]. We therefore determined the LPS aggregate sizes and their distributions in a Zeta sizer, by analyzing the diffusion of the aggregates via measurement of the backscatter signals. Again, rough mutant LPS (LPS from *S. minnesota* Re (R595) and Ra (R60), as well as smooth form (O55:B5)) were analysed.

#### 2.5.1. Results for Deep Rough Mutant LPS R595

**Figure 5:**
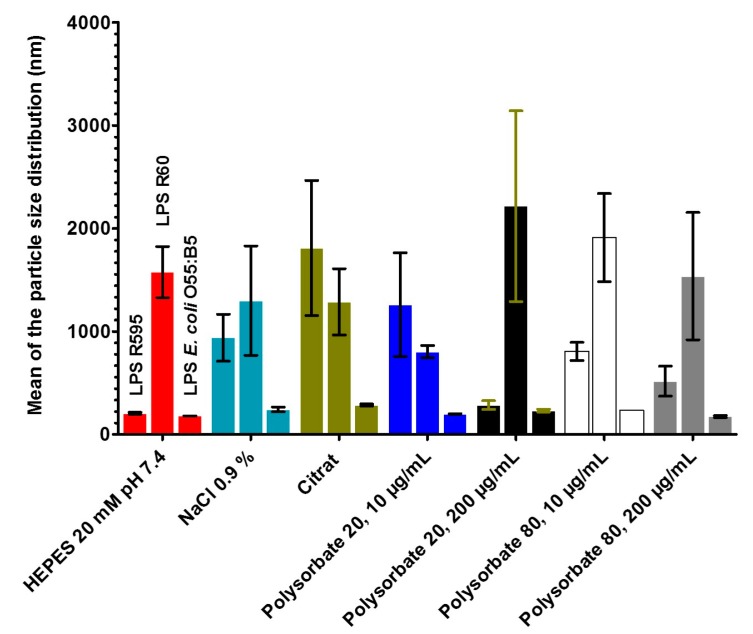
Effect of the formulation on the aggregate size of LPS. The figure shows the particle size for seven different preparations. For each preparation, LPS aggregates are shown as LPS R595 (first bar), LPS R60 (second bar), and LPS *E. coli* O55:B5 (third bar). The error bar results from twofold measurement of TNF-α in the ELISA.

In the following Figure 5 the results are presented for deep rough mutant LPS from *S. minnesota* R595. On the left-hand side, the size distribution is shown, on the right-hand the side polydispersity, i.e., the respective size distributions (see Table 2).

It becomes clear that the LPS sample in HEPES buffer (top left) has lowest values of the peak around 205 nm and a distribution factor of 0.45. Interestingly, the values for the aggregates in NaCl are much higher, and are highest in the citrate formulation. In the two latter samples, also the distributions are broadest. Both polysorbate preparations at 200 μg/mL have rather low sizes, whereas their peak sizes at the smaller polysorbate concentrations are significantly higher.

#### 2.5.2. Results for Rough Mutant LPS Ra with Complete Core Oligosaccharide

In the following, the results are presented for rough mutant LPS from *S. minnesota* R60 (Figure 5 and Table 2).

The results for the rough mutant LPS R60 with complete core oligosaccharide differs considerably from the results for the deep rough mutant LPS. The sizes and their distributions are much more homogenous. In general, the sizes are significantly larger than those from LPS R595. Interestingly, the results for the preparation with polysorbate 20, 200 μg/mL, exhibits the largest sizes, whereas the values for LPS R595 are indicative of very small sizes.

The corresponding data for the wild-type LPS O55:B5 are shown in Figure 5 and Table 2. It is striking that the sizes of the LPS aggregates in the different formulations are considerably lower than for the two rough mutant LPS. Furthermore, similar to LPS R595, the citrate formulation has a highest size (285 nm), which, however, is very low as compared to the former LPS (1811 nm).

Summarized, the data give evidence for a strong dependence on the size distributions from the LPS representing different serotypes. It should be mentioned here, that these results are of course influenced strongly by the facts that the chemical structures of rough mutant LPS are relatively homogenous, whereas wild type forms usually consists of a heterogenous assembly of various part structures, containing an Ra- or Rb-type LPS as bioactive moiety [14].

### 2.6. Size Distribution in Relation to Cytokine Induction in Human Mononuclear Cells

The same samples, which were analysed in light scattering experiments, were added to human mononuclear cells that were obtained after blood separation form healthy donors, and their ability to induce tumor-necrosis-factorα (TNF-α) was measured in an ELISA (MAT). In Figure S2, the results are shown for deep rough mutant LPS from R595 for two concentrations 10 and 1 ng/mL. At the higher concentration, the stimulation values are rather homogeneous, except for the citrate value, whereas at the lower concentration, only the value for polysorbate 80 (10 μg/mL) deviates to lower values.

The results for the rough mutant LPS R60 are given in Figure S3. It can be seen that the absolute TNF-values tend to be lower than for LPS R595. The observation of lower values for citrate at the higher LPS concentration is also observed here, whereas the pattern of the TNF values is more homogeneous, but significantly lower than for LPS R595.

In a similar way, data are presented for LPS S-form O55:B5 (Figure S4). Also, here, the citrate formulation at 10 ng/mL has the lowest activity. Surprisingly, there is a great difference to the cytokine values at 1 ng/mL. The comparison of the three LPS shows that with an increasing length of the sugar chain, which is shortest for LPS Re, longer for LPS Ra, and longest for LPS S-form, the results become more variable.

In another approach, the MAT was performed with two LPS (LPS R60 and O55:B5) and with two pretreatments. Sonicated LPS should produce small, vortexed LPS large aggregate structures. This was performed according to the findings of Komoro et al. [20], who found better reactivity in the pyrogen test and LAL with sonicated LPS preparations. As can be deduced from Figure S5, there is no significant difference in the response of the MAT at both sonicated and vortexed samples.

Summarized, the data presented here do not indicate a general dependence of the biological activities in the MAT assay of different LPS preparations on the respective sizes and size distributions. It should be noted here that the term aggregate size in a sense of a well-defined spherical form for LPS is not well-defined, in particular, for LPS with long saccharide chains, such as S-form (wild-type) LPS.

## 3. Discussion

In a comprehensive analysis, we have performed biophysical analyses of different LPS formulations (detergent, chelating buffer) being assumed to represent the major factors that are mediating the LER-effect. In addition, we also investigated different LPS varying in size and structure, i.e., from wild-type (S-form) over various rough-mutants differing in the size of the LPS core-oligosaccharide (Re-, Rd-, and Ra- mutant LPS). In a first step, we have analyzed the single constituents of the complex compositions of the pharmaceutical, formulations, i.e., citrate, polysorbate 20 and polysorbate 80. The data presented here can serve as the basis for further investigations, in which the complete formulation leading to LER will be tested.

We have found in various test systems, that there are clear changes of different parameters, with variations of the formulation. These data give hints with respect to the occurrence of the LER, in which the LPS backbone structure shows reduced LAL reactivity.

We have analyzed the following physical-chemical parameters, which might be responsible for the LER in LPS formulations:

- Fluidity of their hydrocarbon chains;
- Aggregate size and structure;
- Head group conformation and orientation.

Following this line, we have investigated the:

(i) (i) gel to liquid crystalline phase transition of the hydrocarbon chains of LPS, and with that, the fluidity of the acyl chains, with Fourier-transform infrared spectroscopy (FTIR);
(ii) three-dimensional aggregate structure of LPS by using synchrotron radiation small-angle X-ray scattering (SAXS);
(iii) LPS aggregate sizes by dynamic light scattering and have related these data to the biological activities in the MAT;
(iv) Furthermore, the interaction of LPS with a synthetic anti-LPS peptide Pep19-2.5 was monitored to find out whether differences in head group binding are observed.

It has been shown that the order of the acyl chains (highly ordered = gel phase, less ordered = liquid crystalline phase) influences the bioactivity of LPS and lipid A preparations [21]. Thus, with increasing order (lower fluidity) interaction with target structures such as the factor C in the *Limulus* assay or cell surface receptors, such as CD14 or the TLR4/MD2 complex, are impeded. Therefore, the data for the samples with T_c_ increases (for example, see Figure 1E) should have lowered biological activity in the MAT, because the acyl chains are more rigid. This relates in first line to the polysorbate formulations, which may influence the biological responses. The SAXS data show only small, but significant, changes of the observed aggregate structures on the different formulations. In particular, the existence of highly ordered phases observed for the smooth, as well as rough, mutant LPS R7 and R345 for the polysorbate formulations may give a hint for a masking process, which will be tested in further experiments with the complete formulation system. It has been shown in previous papers [15,17] for lipid A and rough mutant LPS as well as in a recent paper on wild-type LPS [18] that the aggregate structure of LPS is a determinant for its biological activity in the MAT. Thus, non-lamellar, in most cases cubic structures are the bioactive units of LPS. The observation of a shift of the broad scattering range from 0.1 to 0.25 /nm (Figure 2A–C) to 0.13 to 0.35 (Figure 2D) indicates a new, probably highly ordered, phase, for LPS Rd in the polysorbate formulations (interpretation from unpublished results).

Regarding the data from the size distributions presented here, the results indicate for the different LPS mutants/smooth forms quite diverging results. The data are indicative of medium sizes for LPS Re, high sizes for LPS Ra, and low sizes for LPS S-form. For an understanding, the results from studies of LPS morphologies may be useful. It was found that for most rough mutant LPS spherical-like morphologies were reported, by using cryo- and freeze-fracture electron microscopy [22]. In contrast, in LPS with longer sugar chain, in particular S-LPS, membrane vesicles, bilayer disks, and ribbon-like aggregates are found. These data are in accordance with the size distribution that is obtained via ultracentrufigation, in which R-LPS showed size distribution between 100 to 600 nm, whereas for S-LPS, the values were around 50 to 200 nm [22].

It should be noted that in the evaluation of the Zeta sizer measurements, a simple assumption of spherical-like structures would give directly comparable results for the medium sizes. Therefore, the size values for compounds with long saccharide chains, such as S-form LPS, are not the radius of a sphere, but give only a medium value for its non-spherical morphology. Finally, it should be noted that polysorbates—which are added to drug products to inhibit protein aggregation—do not lead to LPS disaggregation at least when administered solely (see Figure S5).

Regarding the comparison of the results from the biological assay at the selected concentrations with those of the three LPS with differences in the saccharide chain lengths in different buffers do not show any systematic dependence of the MAT response with the aggregate sizes and their distributions. Finally, the ITC data of LPS binding to Pep19-2.5 indicate a significantly higher peptide to LPS ratio for binding saturation for the polysorbate formulations, which is indicative of a change in the LPS head group conformation.

Literature data on the one hand explain the LER by increases in aggregate size and stability [23], and, on the other hand, by a decrease down to monomers.

Reich et al. have proposed ‘the supramolecular structure of endotoxin is altered and exhibits only a limited susceptibility in binding of the factor C of Limulus-based detection systems. Although, in our analysis under conditions with reduced complexity (only pure citrate or polysorbates were used, but not a combination therefrom), we observed some changes in the supramolecular assembly and the phase transitions of the acyl chains, in particular when polysorbate is present in the LPS preparations.

Masked endotoxin may adopt a supramolecular conformation not detectable by the LAL test. Schwarz et al. [24] have found for masked endotoxin—as evidenced by the chromogenic endpoint LAL—the expression of pro-inflammatory cytokines and surface activation markers. This is an observation, which we will address in future experiments, in particular by investigating the complete system relevant in LER.

The presented data will form the basis for detailed investigations into the dependence of biophysical parameters of the complete detergent system, in particular on the influence of the 4′-phosphate diglucosamine backbone of the lipid A part of LPS, the recognition structure of LPS by the *Limulus* assay. It is envisaged to continue the investigations by using also the factor C of the *Limulus* assay in recombinant form, and possibly LPS-binding sequences of this, and comparing it with the well-known behavior of anti-endotoxin peptides, such as Pep19-2.5.

From these observations, the following questions seem to be important with respect to the occurrence of the LER: is the lack of endotoxin detection by LAL a problem of LPS in an undetectable, inactive conformation or a failure of the measuring system LAL?

The headgroup conformation, in particular of the 4′-phosphate group in the lipid A part is of central importance. We will perform in a next step an analysis via FTIR by studying the interaction of LPS with rFC and part structures.

Could the change of the LPS conformation into in monomeric form be responsible for the LER? Müller et al. [5] found in the MAT as well as the LAL no biological activity of LPS monomers. There are other publications, however, which come to a completely different conclusion [25]. Therefore, this hypothesis will be in the focus of further studies.

## 4. Materials and Methods

### 4.1. Peptides, Reagents and LPS Formulations

Lipopolysaccharides O55:B5 and O111:B4 from *Escherichia coli* wild-type strains (S-form LPS) were purchased from Sigma (Deisenhofen, Germany), rough mutant LPS Ra strain R60, Rb strain R345, Rd strain R7, and Re strain R595 from *Salmonella minnesota* were extracted from bacteria by phenol/chloroform/petrol ether, according to the protocol of Galanos et al. [26]. For wild-type strains, the chemical structure of LPS consists of the lipid A part, which represents the outer leaflet of the bacterial outer membrane, the oligosaccharide core, and the *O*-antigen, a polysaccharide moiety directing outwards. The chemical structures of the single segments of the LPS molecule from the commonly used wild type strains are—except for the relatively homogenous lipid A moiety (“conservative” motif [1])—not well described, and varies from strain to strain. Usually, the core oligosaccharide, which is bound to the lipid A part, consists of 10 to 12 monosaccharide units, and the subsequent O-antigen has a largely varying polysaccharide chain. Moreover, S-form LPS consists of different fractions, which may have also underacylated lipid A parts [1,14]. The details of these inhomogenities are in most cases unknown except for single analyses as for example performed by Jiao and Galanos [14] for wild-type LPS from *Salmonella abortus equi.*

Rough mutant LPS lack the O-antigen, and have a varying length of the oligosaccharide, Ra with a complete one, and the other mutants having a shorter oligosaccharide in the sequence Rb > Rc > Rd > Re.

The antimicrobial peptide Pep19-2.5 (Aspidasept^®^) with a sequence of GCKKYRRFRWKFKGKFWFWG was synthesized by BACHEM (Bubendorf, Switzerland) with a purity of >95%. All of the other chemicals were from Merck (Mannheim, Germany). Sodium citrate and polysorbate 20 and 80 was purchased from Merck (Mannheim, Germany) and Sigma (Deisenhofen, Germany).

For all of the applied techniques listed below, the LPS samples were prepared as aqueous dispersions in 20 mM HEPES pH 7.4, 30 mM sodium citrate pH 4.0, polysorbate 20 and 80, the latter each at 10 and 200 μg/mL. The latter concentration corresponds to values below and above the critical micellar concentration, respectively. LPSs were suspended directly in buffer by extensively vortexing, sonicated in a water bath at 60 °C for 30 min, cooled down to 5 °C, and subjected to three cycles of heating and cooling from 60 to 5 °C. After that, the lipid samples were stored for at least 24 h at 4 °C before performing the measurements.

### 4.2. Acyl Chain Melting Behavior by Fourier-Transform Infrared Spectroscopy

The infrared spectroscopic measurements were performed on a FTIR spectrometer IFS-55, from Bruker (Karlsruhe, Germany). The lipid samples were placed in a CaF_2_ cuvette separated by a 12.5 mm thick teflon spacer. Temperature-scans were performed automatically in the range from 10 to 65–80 °C with a heating rate of 0.6 °C min^−1^. Every 3 °C, 200 interferograms were accumulated, apodized, Fourier transformed, and converted to absorbance spectra. The phase behaviour was monitored by using the peak position of the symmetric stretching vibration ν_s_ (CH_2_) in the wavbenumber range 2850 to 2853 cm^−1^. The phase transition temperature *T_c_* can be determined by taking the midpoint of the intersection of the tangents of the curve in the gel phase with that of the inflection point of the transition range, and the intersection of the latter with the tangent of the curve in the liquid crystalline phase.

### 4.3. Aggregate Structure Determined by Small-Angle X-ray Scattering (SAXS)

The X-ray scattering measurements were performed on the X33 beamline of the European Molecular Biology Laboratory (EMBL) outstation at HASYLAB on the storage ring PETRA of the Deutsches Elektronen Synchrotron (DESY) at Hamburg [27].

Briefly, scattering patterns in the range of scattering vector 0.05 < s < 1 nm^−1^ (s = 2 sin *θ*/*λ*, 2 *θ* is the scattering angle and λ the wavelength = 0.15 nm) were recorded, with exposure times of 1min using an image plate detector with online readout (Mar345; Marresearch, Norderstedt, Germany). Further details concerning the data acquisition and evaluation have been described previously [12]. In the diffraction patterns that are presented below, the logarithm of the diffracted intensities I(s) is plotted versus s. The X-ray spectra were evaluated using standard procedures [11], which allow for assigning the spacing ratios of the diffraction maxi-ma to defined three-dimensional structures of the endotoxin: detergent samples.

Structures occuring for endotoxins comprise lamellar (L) phases with spacing ratios lying at equidistant positions and nonlamellar phases like cubic (Q) and inverted hexagonal (HII) that are characterized by square root spacing ratios [28].

### 4.4. Binding Affinity of LPS to Pep19-2.5 via Isothermal Titration Calorimetry (ITC)

The interaction of the peptide Pep19-2.5 with LPS in various formulations was analyzed by microcalorimetric measurements in the ITC200 (GE Healthcare, Munich, Germany), as recently described [19]. For this, 1 mM (2.71 mg/mL) Pep19-2.5 in different formulations was titrated into 430 μg LPS from *E. coli* O55:B5 and the measured enthalpy changes (∆H) were recorded versus time and the peptide: LPS concentration ratio.

### 4.5. Particle Size Measurements by Dynamic Light Scattering on a Zeta Sizer

Dynamic light scattering of the particle sizes of LPS aggregates was performed in different formulations, by measuring the diffusion velocity in a Malvern Zeta sizer Nano (Malvern, Herrenberg, Germany). The method is based on the measurement of the diffusion of small particles according to the Stokes-Einstein equation D = μ × k_B_ ×T (μ = mobility of the particles, k_B_ = Boltzmann constant), measuring the back-scattering, and calculating the autocorrelation function. Each particle scatters the light to the detector, and the fluctuations of the scattering intensity is smaller for large than for small particles.

In detail, the LPS samples were measured for 3 min in a fixed laser position of 173° (backscattering), relative to the incident laser beam. The measured intensities were correlated over time and analysed by a multiple exponential, non-negative least square fit to obtain relative intensities for the different particle sizes. The LPS samples at concentrations of 10 μM were dispersed in following formulations: 20 mM Hepes buffer at pH 7, 0.9% NaCl, 30 mM citrate, 10 and 200 μg/mL polysorbate 20, respectively, and 10 and 200 μg/mL polysorbate 80. The samples were prepared by sonication and temperature-cycled between 20–60 °C, and were stored at room temperature.

### 4.6. Stimulation of Human Mononuclear Cells

Mononuclear cells (MNC) were isolated from heparinized blood samples that were obtained from healthy donors, as described previously [15]. The cells were resuspended in medium (RPMI 1640), and their number was equilibrated at 5 × 10^6^ cells/mL. For stimulation, 200 μL MNC (1 × 10^6^ cells) was transferred into each well of a 96-well culture plate. The LPS formulations were preincubated for 30 min at 37 °C and were added to the cultures at 20 μL per well. The cultures were incubated for 4 h at 37 °C with 5% CO_2_. Supernatants were collected after centrifugation of the culture plates for 10 min at 400× *g* and stored at 20 °C until immunological determination of tumor necrosis factor alpha (TNF-α), carried out with a sandwich enzyme-linked immunosorbent assay (ELISA) using a monoclonal antibody against TNF (clone 6b; Intex AG, Basel, Switzerland), and described previously in detail [19].

## 5. Conclusions

In conclusion, the current work has laid out a number of analytical approaches to study the LPS system and provide insight into the structural changes that the LPS might be going through, subsequently leading to the LER effect. The next steps to be investigated will be the combination of chelating buffers and polysorbate and study their individual impact on the LER effect, which is most relevant for pharmaceutical preparations. Also, the study in various (chelating) buffers (e.g., histidine, citrate, acetate, succinate, phosphate, etc.) on structural details, in the presence of polysorbate 20 and 80, respectively. In addition, the presence or absence of divalent cations, such as Mg^2+^ and Ca^2+^, which are necessary for the formation of defined and complex negatively charged LPS aggregates, as well as the pH-value seems to be of outmost importance for the understanding of the LER-effect on a molecular level. Finally, the same holds true for surfactant concentrations from 0.1 to 2 mg/mL, i.e., in a pharmaceutical relevant range.

## Figures and Tables

**Table 1:** Thermodynamic parameters of the interaction of LPS from *E. coli* 055:B5 with the synthetic anti-endotoxin peptide (Pep19-2.5) formulations. PS: polysorbate.

Thermodyamic Parameters	LPS 055:B5 and Pep19-2.5 Dissolved in the Same Medium					LPS 055:B5 Dissolved in Polysorbates Pep19-2.5 Dissolved in Water			
	LPS in HEPES	PS20 10 µg/mL	PS20 200 µg/mL	PS80 10 µg/mL	PS80 200 µg/mL	PS20 10 µg/mL	PS20 200 µg/mL	PS80 10 µg/mL	PS80 200 µg/mL
Mass ratio (Peptide/LPS)	0.25	0.29	0.38	0.39	0.34	0.33	0.32	0.40	0.37
Kd (nM)	862	529	225	104	78	200	218	46	261
ΔH (kJ/mol)	−67.31	−59.82	−45.64	−58.35	−56.58	−48.49	−49.14	−43.00	−48.60
ΔS (kJ/mol·K)	−0.10	−0.07	−0.02	−0.05	−0.05	−0.03	−0.03	0.01	−0.03

**Table 2:** Polydispersity index (PDI) for lipopolysaccharides aggregates in different formulations.

Formulation	Polydispersity Index (PDI)		
	LPS R595	LPS Ra	LPS O55:B5
HEPES 20 mM pH 7.4	0.449 ± 0.012	0.983 ± 0.029	0.436 ± 0.004
NaCl 0.9%	0.935 ± 0.112	1.000 ± 0.000	0.524 ± 0.030
Citrate 30 mM pH 4.0	1.000 ± 0.000	1.000 ± 0.000	0.536 ± 0.018
Polysorbate 20, 10 μg/mL	1.000 ± 0.000	0.934 ± 0.073	0.514 ± 0.028
Polysorbate 20, 200 μg/mL	0.966 ± 0.058	0.966 ± 0.058	0.506 ± 0.059
Polysorbate 80, 10 μg/mL	0.911 ± 0.083	1.000 ± 0.000	0.463 ± 0.002
Polysorbate 80, 200 μg/mL	0.814 ± 0.050	1.000 ± 0.000	0.469 ± 0.056

## Supplementary Materials

The following are available online at www.mdpi.com/1422-0067/18/12/2737/s1.
