# Supplementary material for: Biophysical Analysis of Lipopolysaccharide Formulations for an Understanding of the Low Endotoxin Recovery (LER) Phenomenon
Source: Int J Mol Sci. 2017 Dec 16;18(12):2737. doi: 10.3390/ijms18122737 (PMC5751338; doi:10.3390/ijms18122737)
Supplement: Supplementary file 1 [file ijms-18-02737-s001.docx]

**Supplementary Materials**


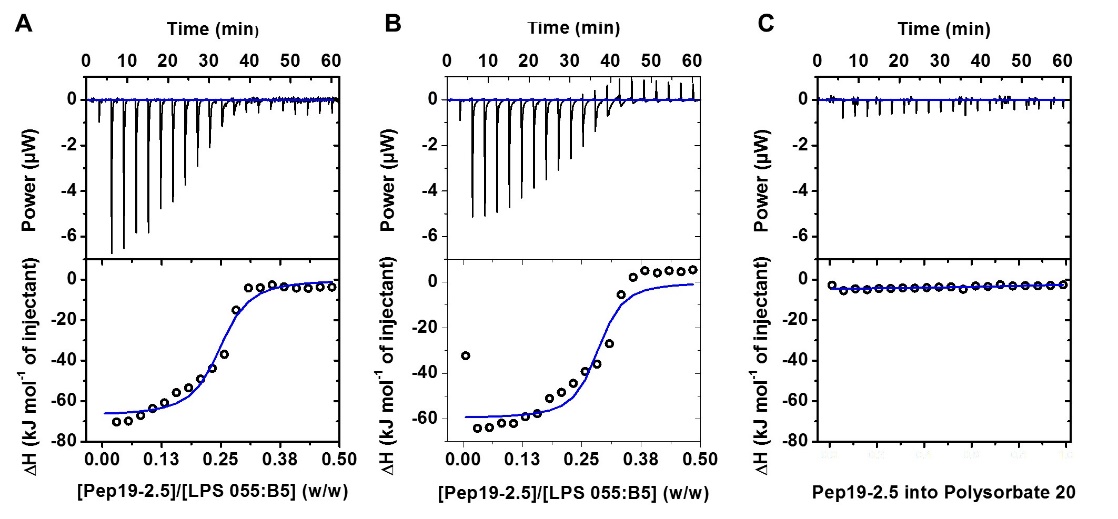


**Figure S1**. *Isothermal titration calorimetric measurements of the interaction between Pep19-2.5 and LPS from E. coli O55:B5*. Compounds were dissolved in 20 mM HEPES pH = 7,4 (A) or Polysorbate 20 (10µg/ml) (B); as a control, Pep19-2.5 were titrated into polysorbate 20 (10 µg/ml) (C). 1 µl of Pep19-2.5 (1.0 mM) were titrated 20 times into 250 µl of LPS from *E. coli* O55:B5 (430 µg/ml).


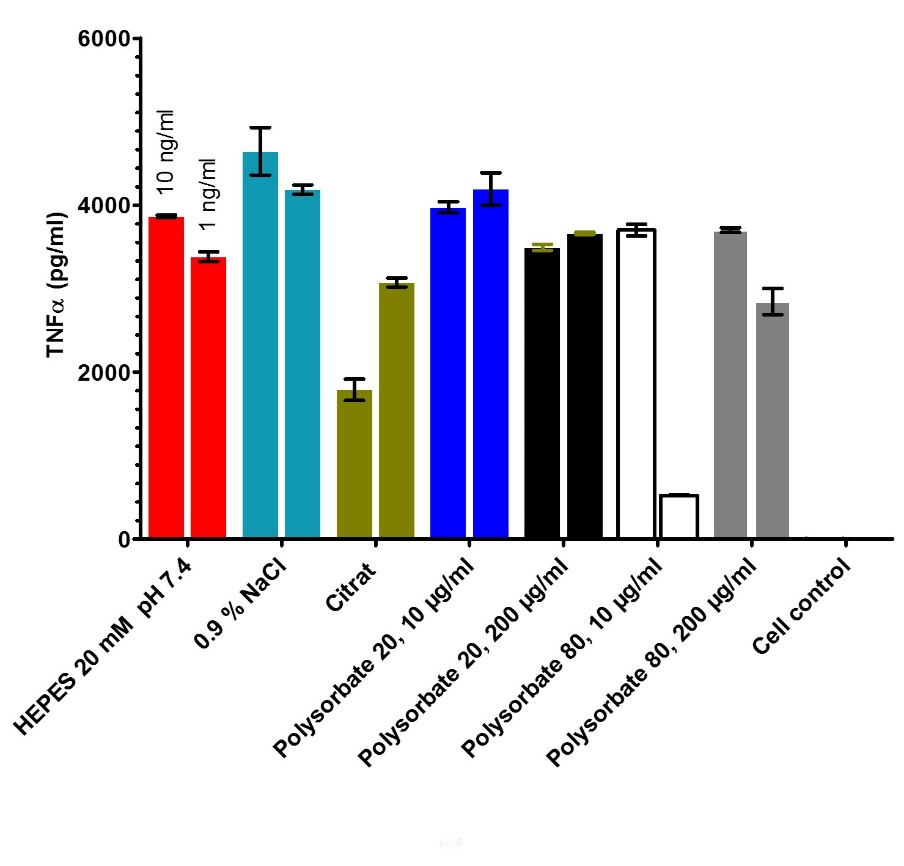


**Figure S2**. Secretion of TNFα of human mononuclear cells induced by deep rough mutant LPS R595 in different formulations.


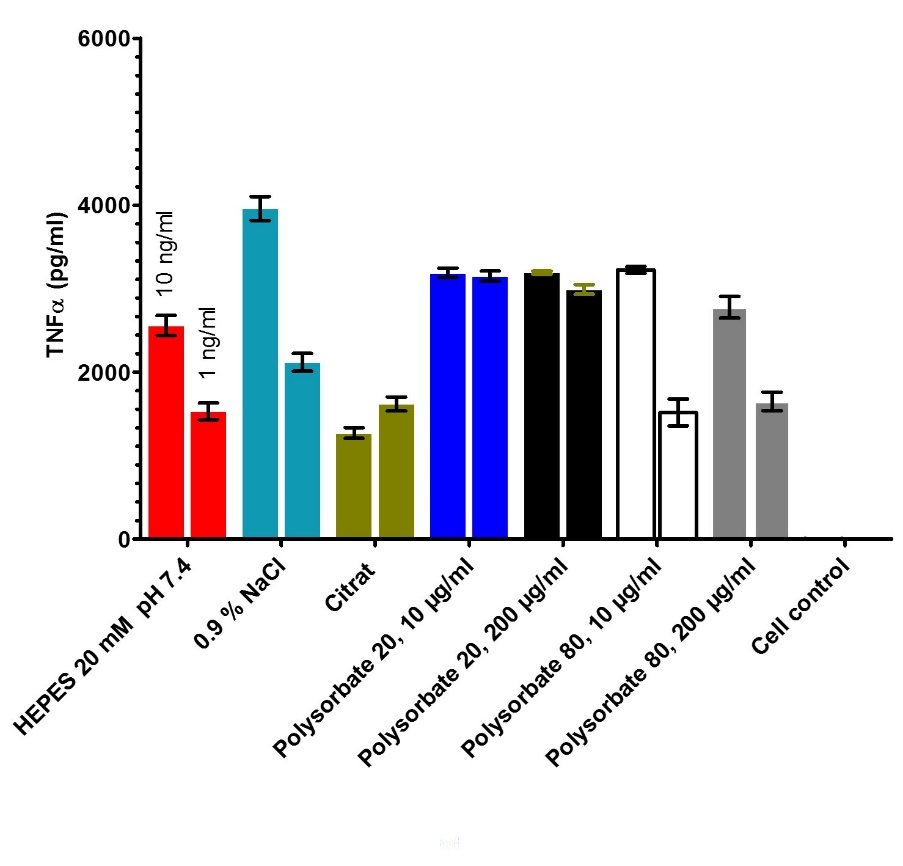


**Figure S3**. Secretion of TNFα of human mononuclear cells induced by rough mutant LPS R60 in different formulations.


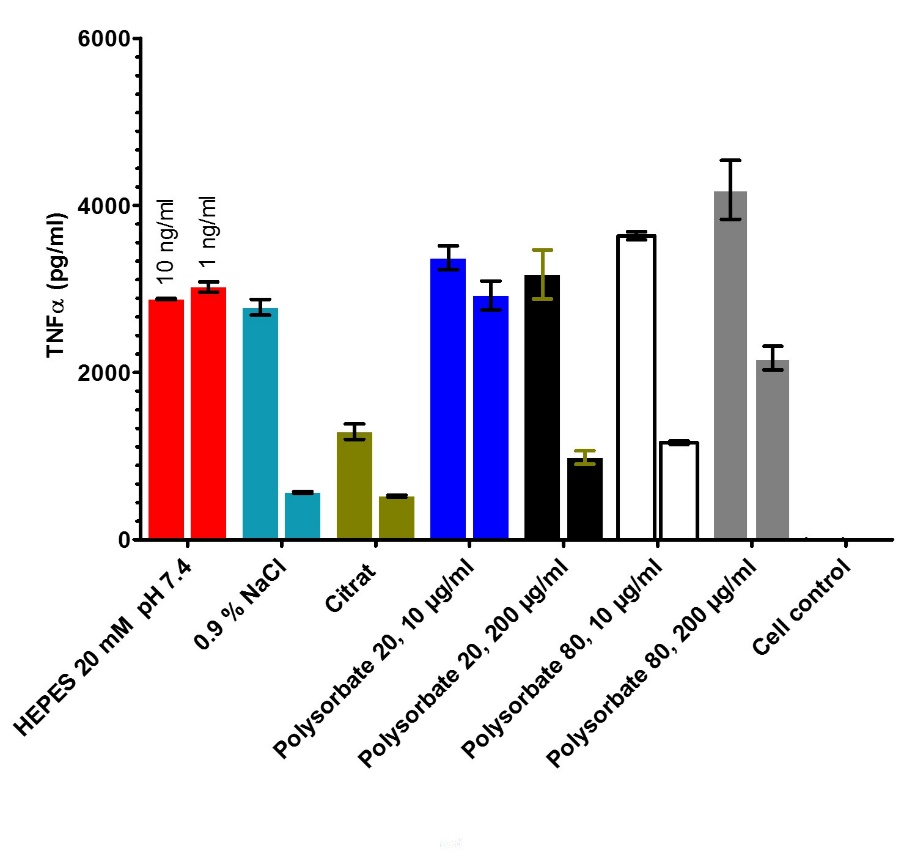


**Figure S4**. Secretion of TNFα of human mononuclear cells induced by smooth form LPS O55:B5 in different formulations.


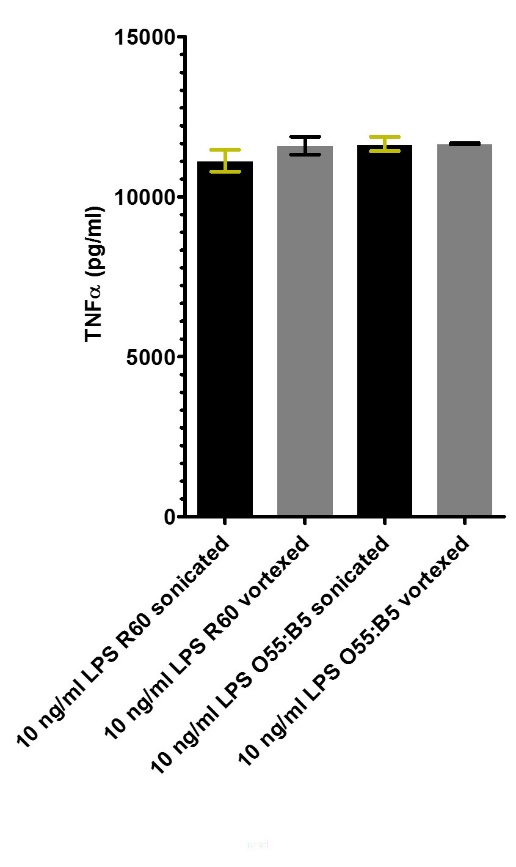


**Figure S5**: Secretion of TNFα of human mononuclear cells induced by the rough mutant LPS R60 and the smooth form LPS O55:B5 with two pretreatments (sonicated and vortexed).
